# Evidence from neglect dyslexia for morphological decomposition at the early stages of orthographic-visual analysis

**DOI:** 10.3389/fnhum.2015.00497

**Published:** 2015-10-15

**Authors:** Julia Reznick, Naama Friedmann

**Affiliations:** Language and Brain Lab, Tel Aviv UniversityTel Aviv, Israel

**Keywords:** morphology, morphological decomposition, reading, neglect dyslexia, Hebrew

## Abstract

This study examined whether and how the morphological structure of written words affects reading in word-based neglect dyslexia (neglexia), and what can be learned about morphological decomposition in reading from the effect of morphology on neglexia. The oral reading of 7 Hebrew-speaking participants with acquired neglexia at the word level—6 with left neglexia and 1 with right neglexia—was evaluated. The main finding was that the morphological role of the letters on the neglected side of the word affected neglect errors: When an affix appeared on the neglected side, it was neglected significantly more often than when the neglected side was part of the root; root letters on the neglected side were never omitted, whereas affixes were. Perceptual effects of length and final letter form were found for words with an affix on the neglected side, but not for words in which a root letter appeared in the neglected side. Semantic and lexical factors did not affect the participants' reading and error pattern, and neglect errors did not preserve the morpho-lexical characteristics of the target words. These findings indicate that an early morphological decomposition of words to their root and affixes occurs before access to the lexicon and to semantics, at the orthographic-visual analysis stage, and that the effects did not result from lexical feedback. The same effects of morphological structure on reading were manifested by the participants with left- and right-sided neglexia. Since neglexia is a deficit at the orthographic-visual analysis level, the effect of morphology on reading patterns in neglexia further supports that morphological decomposition occurs in the orthographic-visual analysis stage, prelexically, and that the search for the three letters of the root in Hebrew is a trigger for attention shift in neglexia.

## 1. Introduction

One of the intriguing questions in the cognitive psychology and neuropsychology of reading relates to how we read words like “segmentation,” “absolutely,” “smiling,” or “kangaroos.” If such morphologically complex words are represented in the orthographic lexicon in a decomposed form, access to the lexicon should use morphologically decomposed codes. To allow for such access, a pre-lexical stage of morphological decomposition is required.

Word-based neglect dyslexia (neglexia), a reading deficit in which letters on one side of the word are neglected, provides an interesting opportunity to examine the process of morphological decomposition. Because neglexia occurs at the stage of the orthographic-visual analysis of words, an effect of the morphological structure of words would indicate that such early morphological decomposition occurs at the stage of orthographic-visual analysis, and would enable the examination of the characteristics of this early morphological decomposition.

### 1.1. Morphological representation and processing of written words

The first stage of the reading process is a stage of visual-orthographic analysis, according to the model we assume here, the dual route model for word reading (Morton and Patterson, 1980; Newcombe and Marshall, 1981; Coltheart, 1984, 1985; Marshall, 1984; Coltheart et al., 1993, 2001; Ellis and Young, 1996; Jackson and Coltheart, 2001). This first stage is responsible for recognizing the abstract identity of the letters in the word, for encoding the relative position of letters in the word, and for binding the letters to the words they appear in. The output of the orthographic-visual analysis then enters the orthographic input lexicon, possibly through an orthographic input buffer^1^. The orthographic input lexicon contains the written form of words, and reading proceeds by a search for a word in this lexicon that matches the input information regarding the identity and position of the letters. The information from the orthographic-visual analyzer is also transferred to the other reading route—the sublexical route, which is based on grapheme-to-phoneme conversion, and enables the reading of unfamiliar words and of non-words.

There are three main types of approaches to the way in which morphologically complex words are represented in the orthographic input lexicon, from which different approaches are derived for explaining morphological decomposition at the pre-lexical stage.

According to one approach, no morphological decomposition of morphologically complex words occurs pre-lexically (e.g., Manelis and Tharp, 1977; Lukatela et al., 1980, 1987; Butterworth, 1983; Giraudo and Grainger, 2000, 2001). Nonetheless, some of the researchers who hold this full-listing view suggest that morphology does act as an organizing factor of lexical representations in the lexicon (Lukatela et al., 1980, 1987), or alternatively, that morphological decomposition occurs at a post-lexical stage (Giraudo and Grainger, 2000, 2001). There are also researchers who completely reject the relevance of morphology to the processing and representation of written words, and claim that the morphological effects that have been found in studies are no more than an expression of the ensemble of associations that exist between words (Seidenberg and McClelland, 1989).

According to the opposite approach, morphological decomposition of morphologically complex words is a necessary part of the process of accessing their lexical representations (e.g., Taft and Forster, 1975; Rastle et al., 2004; Taft and Kougious, 2004; Longtin and Meunier, 2005; Crepaldi et al., 2010, and see Amenta and Crepaldi, 2012, for a review). According to one of these models, words are stripped of their affixes pre-lexically and the stem is used as a lexical unit of access (Affix-Stripping Model, ASM, Taft and Forster, 1975; Taft, 1979, 1981). Another model that postulates obligatory morphological decomposition suggests that word access occurs through the activation of the morphemes that the word is composed of (the Interactive Activation Model, IAM, Taft, 1994).

An intermediate approach, the dual-access approach, postulates that the lexical units of access can be either morphemes and/or whole words (Baayen et al., 1997; Diependaele et al., 2009). Whereas some assume there to be a parallel activation of both the whole-word and the morpheme routes (e.g., Meta Model, Schreuder and Baayen, 1995), others determine the method of access (one route or both in parallel) according to the characteristics and morphological structure of the target word (Augmented Addressed Morphology Model, AAM, Laudanna and Burani, 1985; Burani and Caramazza, 1987; Caramazza et al., 1988; Chialant and Caramazza, 1995; Traficante and Burani, 2003). According to the AAM, both the whole word units and the morpheme units are used to access the lexicon, in which the words are stored in a morphologically decomposed form (at least the regularly inflected words). Thus, according to this approach, morphological decomposition is optional.

A further debate relates to whether early morphological decomposition relies solely on structural, morpho-orthographic pre-lexical analysis (identification of units that enable morphological decomposition) or whether it is based on lexical information (e.g., whether a certain combination of morphemes forms an existing word; see also Meunier and Longtin, 2007).

Whereas most studies of morphological decomposition asked these questions of whether decomposition is obligatory and what its nature is through the assessment of normal reading, mainly using priming tests, the current study approaches these questions from a novel perspective: that of reading in peripheral dyslexia. We examine whether morphological decomposition occurs in the process of lexical access and when it occurs, by studying the effect of the morphological structure of words on reading in neglect dyslexia (neglexia). Given that neglexia is a deficit at the pre-lexical stages of reading, if the morphological structure is found to affect reading in neglexia, this will provide evidence for morphological decomposition, and locate it before the lexicon. We will also assess whether this morphological decomposition is affected by lexical and semantic factors and what guides this early decomposition. This study was conducted in Hebrew, a morphologically rich language, and the following section surveys what is known about the effect of morphology on reading in Hebrew.

### 1.2. Representation and processing of morphologically complex words in hebrew

Hebrew is a Semitic language with an alphabetic orthography, read from right to left. As a language with Semitic morphology, most Hebrew words are composed of a tri-consonantal root and affixes. Verbs, nouns, adjectives, and prepositions can include inflectional morphology, and inflect for gender, number, and possessor/genitive; verbs also inflect for tense and person. As for derivational morphology, verbs, nouns, and adjectives are created from a root and a template: verbs are formed in a verbal template called “binyan” (Arad, 2005; Arad and Shlonsky, 2008), nouns and adjectives are inserted into a nominal template (“mishkal”). The inflectional and derivational morphemes may be vowels or consonants. They are not only linearly added to the beginning or end of the root, but may be interwoven, with the root and affixes appearing alternately. The vowels and consonants of one morpheme (word pattern) can appear between the letters of another morpheme (the root), so the letters of the root can be non-adjacent. Thus, affix letters can appear before the root, in the middle of the root, or after it, namely, in the beginning, middle, or end of the word, and often in several positions in the same word (see Table 1 for examples).

**Table 1:** **Examples for inflected and derived words in Hebrew for the root 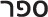, SPR. The root appears in 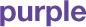, inflectional morphology in 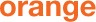, derivational in 
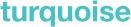
. The root meanings relate to stories, numbers, and hair-cutting**.

**Inflection**				**Derivation**			
**Hebrew**	**Transliteration**	**Transcription**	**Translation**	**Hebrew**	**Transliteration**	**Transcription**	**Translation**
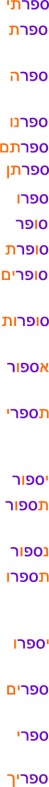	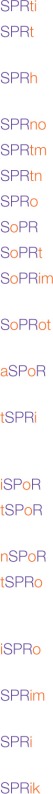	safarti	counted-1sg	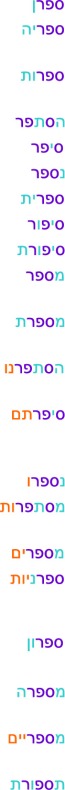	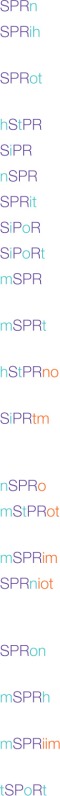	safran	librarian-mas
		safart, safarta	counted-2sg-fem counted-2sg-mas			sifriya	library
		safra, sifra	counted-3sg-fem, her book, digit			sifrut, sfarot, saparut	literature, digits, hairdressing
		safarnu	counted-1pl			histaper	got-a-haircut (cut-hair-refl)
		safartem	counted-2pl-mas			siper	told, cut hair
		safarten	counted-2pl-fem			nispar, nesaper	was-counted, tell-fur-1pl
		safru	count-past-3pl			saparit, sifriyat	hairdresser-fem, library-of
		sofer	counts-mas, author-mas			sipur	story
		soferet	counts-fem, author-fem			siporet	fiction
		sofrim	count-pres-pl-mas, author-pl-mas			mispar, (mesaper)	number, (can also be inflection: tells-3sg-mas)
		sofrot	count-pres-pl-fem, author-pl-fem			misperet	scissor-kick
		espor	count-fut-1sg			histaparnu	We-got-a-haircut (cut-hair-refl-1pl)
		tesapri, tisperi	cut-hair-fut-2sg-fem tell-fut-2sg- fem, count-fut-2sg-fem			sipartem	told-past-2pl, cut hair-past-2pl
		yispor	count-fut-3sg-mas			nisperu	were-counted
		tispor	count-fut-2sg-mas			mistaprot	getting-a-haircut-pres-pl-fem
		nispor	count-fut-1pl			misparim	numbers
		tisperu, tesapru	count-fut-2pl, cut-hair-fut-2pl, tell-fut-2pl			safraniyot	librarian-fem-pl
		yesapru	cut-hair-fut-3pl tell-fut-3pl			sifron	booklet
		sfarim, saparim	books, barbers			mispara	barbershop
		sifri, sapri	my-book, tell-imperative-fem-sg			misparayim	scissors
		sfarayix	your-books			tisporet	haircut

*Some of the words have additional readings, we chose the main ones for simplicity*.

All letters in Hebrew can be part of the root, 12 letters can also serve as part of inflectional or derivational affix, whereas 10 other letters cannot be part of any affix. Some letters can serve as affixes only in the beginning of the word (e.g., 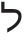, 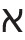), and other letters can appear as affixes before, within, and after the root (e.g., 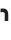, 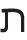), or both before and after the root (e.g., 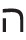)^2^.

In languages with an alphabetic orthography and a linear morphology, the organization of the lexicon reflects, among other things, the orthographic similarity between the words. In Hebrew, the words are thought to be organized according to their morphological structure in the lexicon (Frost et al., 2005; Frost, 2012), and hence, words like 
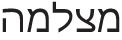
 (mCLMh, *maclema*, camera)^3^ and 
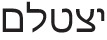
 (iCţLM, *yictalem*, will-be-photographed), which share a root (CLM), are thought to be represented adjacently in the lexicon, even though they are not very similar orthographically (see also the words 
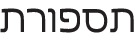
 and 
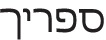
 in the bottom of Table 1).

Findings from normal reading of Hebrew, mainly from studies by Avital Deutsch, Ram Frost, and their colleagues (e.g., Frost et al., 1997; Deutsch et al., 1998, 2000) indicate that the root morpheme mediates access to words in the lexicon, as words prime other words with the same root, regardless of semantic relation, and more so than orthographically similar words. Nouns prime nouns with the same root. For verbs, both the root, and the verbal template show priming effects, suggesting that the affix also has a mediating role in lexical access (Deutsch et al., 1998). Even a root that is not an existing word in itself mediates the identification of words that are derived from it (Frost et al., 1997). Morphologically complex non-words that are composed of an existing root and a verbal template also undergo decomposition (Deutsch et al., 1998). Additional findings indicate that the speed of decomposition is similar when the root's consonants are joined or dispersed (Feldman et al., 1995; Frost et al., 1997), providing evidence of the non-linear nature of word scanning in Hebrew.

Morphological decomposition in Hebrew is disrupted in the case of defective roots, which do not include three consonants. The addition of a random consonant to these verbs, which creates a pseudo-root, re-establishes morphological decomposition (Frost et al., 2000a), indicating that the decomposition mechanism in Hebrew does not require an existing root to decompose the verb to its constituents. This finding clarifies that morphological decomposition is guided by the word's structure and not by lexical factors such as whether the root exists in the lexicon.

In Hebrew, there are many words that are morphologically related but not semantically related. Bentin and Feldman (1990), Frost et al. (1997), and Frost et al. (2000b) used this fact to show that morphological effects can occur in the absence of semantic relations between the words in Hebrew. Frost et al. (1997) used a masked priming task and found that priming effects for morphologically related words were almost identical for semantically related and unrelated words. Bentin and Feldman (1990) used delayed repetition priming at long lags, and reached similar conclusions. They compared semantically related pairs (with and without morphological relation) and morphologically related pairs (with and without semantic relation), and showed that words that share the root but are unrelated semantically show significant repetition effects even at long lags, whereas semantic associations showed priming only at short lags. Frost et al. (2000b) used a cross-modal priming task and also found a strong morphological effect beyond the semantic and phonological relations between words. Morphological priming occurred in their task even when there was morphological (both are derived from the same root), but no semantic relation between the prime and the target. Frost et al. (2000b) concluded that morphological priming cannot be accounted for by semantic and phonological factors alone. The broader implications of their study are that the source of the priming effect reflects morphological processes that are not constrained by semantic factors. Furthermore, the results pertain to the lexical organization of words in Hebrew, and probably other Semitic languages: these results suggest that words are organized by a morphological dimension.

It is interesting to compare these conclusions from Hebrew to conclusions drawn from non-Semitic languages like English and Italian. Some studies (e.g., Marslen-Wilson et al., 1994) found evidence for morphological decomposition of semantically transparent forms, but not of semantically opaque ones. In other studies (e.g., Feldman and Soltano, 1999), morphological facilitation was insensitive to semantic transparency in early stages of reading, and semantics became relevant later. Yet other studies of English report, like Hebrew, a non-semantic morphological priming effect. For example, Kempley and Morton (1982) found this effect in long term priming of spoken words presented in noise. They found a strong facilitation from words inflectionally related to the test word (e.g., reflect/reflected). Importantly, there was no facilitation from semantically related words that were not morphologically related, in words with irregular inflection (e.g., lost/loses), suggesting that the facilitation was morphological rather than semantic.

Hence, studies on normal reading of morphologically complex words in Hebrew indicate that this morphological decomposition is a non-semantic, structural process, which extracts the roots from nouns and verbs, and applies even for morphologically complex non-words. In this study, we will examine the stage at which morphological decomposition occurs by studying the effect of morphological structure on the reading of people with a pre-lexical deficit in visual-orthographic analysis—neglexia.

### 1.3. Neglexia

Neglect dyslexia is a type of dyslexia in which one side of the stimulus is neglected, usually the left side. The literature reports neglect dyslexia at the word level and at the text level (de Lacy Costello and Warrington, 1987; Patterson and Wilson, 1990; Haywood and Coltheart, 2001; Friedmann and Nachman-katz, 2004; Nachman-katz and Friedmann, 2007; Vallar et al., 2010; Friedmann et al., 2011). This study focuses on acquired neglect dyslexia at the word level, which we term *neglexia*. Neglexia is manifested in neglect errors in word reading, i.e., omissions, substitutions, and additions of letters, on one side of the target word. Neglexia belongs to the group of peripheral dyslexias, caused by a deficit at the early, pre-lexical stages of orthographic-visual analysis of written words (Caramazza and Hillis, 1990; Riddoch, 1990; Ellis and Young, 1996; Haywood and Coltheart, 2001).

#### 1.3.1. The effect of morphology on reading in neglexia

Although many studies explored in depth many aspects of neglexia (see, for example, Ellis et al., 1987; Riddoch, 1990; Ellis et al., 1993; Haywood and Coltheart, 2001), only few studies evaluated the role of morphology in neglexia, and neglexia is often thought to be affected by spatial, rather than morphological, factors. For example, Caramazza and Hillis (1990) concluded that “the representation computed at the level of the grapheme description does not contain morphological structure” (p. 420). However, the performance of NG, the participant with right-neglect they describe in that article (summarized in their Table 11, p. 420) was actually affected by the morphological structure of the target words. She made significantly more errors on the right side in words that end with suffixes (222/383, 58%) than in words in which the same stems appeared on the right side (with no affixes) (122/383, 32%; χ^2^ = 52.77, *p* < 0.0001).

Arduino et al. (2002) examined the effect of two morphological measures on oral reading in neglexia: lexical frequency of the words' morphological components and the morphological complexity of the target non-word. They found that some (but not all) the participants were affected by the frequency of the root and the suffix, reading words in which the morphological components were of high frequency better than words with the same frequency in which the morphological components had lower frequency. Similarly, some (but not all) the participants read morphologically complex non-words that included a real root and a real suffix better than morphologically simple non-words. These findings (and see also Vallar et al., 2010, for a review) indicate that the morphological structure of the target word affects the reading of some individuals with neglexia. Arduino et al. (2002, 2003) and Marelli et al. (2013) discuss the morphological effect in neglexia and suggest that they result from an interaction of lexical knowledge with the residual perceptual analysis of the neglected portion of the stimulus that is available to the neglexic reader.

In the current study we aim to further explore, using this effect of morphological structure on reading in neglexia, the stage at which morphological decomposition occurs, the mechanism by which neglect errors are affected by the morphological structure, and the nature of morphological decomposition at the early stage of reading. The general rationale was that given that neglexia is a very early deficit in the process of single word reading, then if the morphological structure of the target word affects reading in neglexia, which could not be ascribed to lexical feedback, this would indicate that morphological decomposition occurs at an early stage of the reading process. We will further explore the nature of the effect of morphology by examining whether perceptual effects such as word length and letter forms are sensitive to morphology, which would establish the early stage at which this effect occurs. We will then assess the extent to which lexical and semantic factors modulate the effect of morphology on neglect errors. We will do so by assessing the morphological effects on neglect errors in pseudo roots and pseudo affixes. Namely, we will test the rates of neglect errors of components that can, structurally, be roots/affixes in the target word, but are not real roots/affixes, and compare them to real roots and affixes. We will also examine whether the erroneous responses preserve the semantic or morpho-lexical features of the target word. If these lexical and semantic factors do not have an effect on neglect errors, this would further support the notion that morphological decomposition is active during the early stage of visual-orthographic analysis, and would rule out a mechanism according to which morphology affects neglect errors by way of feedback from later, lexical, stages.

## 2. Method

### 2.1. Participants

Seven individuals with neglexia at the word level following brain damage participated in this study (Table 2). All participants had acquired neglexia, as diagnosed using standard language tests (the Hebrew versions of the WAB, Kertesz, 1982; Hebrew version by Soroker, 1997; or the ILAT, Shechther, 1965) conducted when they were admitted to the rehabilitation centers. Six of them had left-sided neglexia, and one had right-sided neglexia. None of the participants had syntactic or morphological problems (according to the WAB and the ILAT). Five of the participants were native speakers of Hebrew (one of them was bilingual), and two participants (T. and K.) had been living in Israel and speaking and reading Hebrew for over 40 years at the time of their stroke. As shown in Table 2, some of the participants had a general visuo-spatial neglect, as assessed by the Behavioural Inattention Test (BIT, Wilson et al., 1987), and some also had neglect at the text/sentence level.

**Table 2:** **Background information on the participants**.

**Participant**	**Neglect Type**			**Gender**	**Age**	**Language**	**Education Years**	**Etiology**	**Hemiplegia**
	**General**	**Text**	**Word**						
B.		Left	Left	Female	79	Hebrew	10	Right CVA- subacute infarct in right MCA territory	Left hemiplegia
H.	Left	Left	Left	Female	43	Hebrew	14	Right CVA hemorrhage-right basal and intraventricular	Left hemiplegia, left hypoesthesia
Z.	Left		Left	Male	60	Hebrew, Italian	12	Right CVA	Left hemiplegia
C.		Left	Left	Male	57	Hebrew	12	Right CVA-acute infarct internal capsule	Left hemiplegia
T.			Left	Female	65	Hebrew, Polish	12	Right CVA	
K.			Left	Male	62	Hebrew, French	12	Right CVA hemorrhage-right basal and intraventricular	
R.			Right	Male	60	Hebrew	12	3 years after removal of fronto-parietal tumor. Recent removal of tumor in the left caudate.	Right hemiplegia

### 2.2. Procedure and material

The participants read aloud a list of single words that end or start with derivational or inflectional affixes (Tiltan Test for Neglexia, Friedmann and Gvion, 2003), with no time limit. If the participant gave several responses for the same target word, only the first response was included in the analysis. Importantly, the words in the list were selected so that a left and/or right sided neglect error on each of these words creates other existing words. The words were presented to the participants as a list, one above the other, in the middle of an A4 white page. Different participants read different numbers of words which were relevant for further analyses, ranging between 88 and 163 words. (these differences resulted from some of the patients not being available for more than one meeting, and the difference in their severity of impairment and degree of frustration). Across the list, the same root appeared only once (except for one root that appeared in three morphological templates), and the morphological inflections and derivations of the target words varied so that the same morphological template (derivational + inflectional) repeated four times at most, and most of the morphological templates appeared only once or twice in the list. The protocol has been approved by the Tel Aviv University Ethics committee (Department of Psychology), and the participants signed written informed consent forms, which were read and explained to them.

### 2.3. Data analysis

#### 2.3.1. Potential for lexical errors

Neglect dyslexia causes letter omissions, letter substitutions, and letter additions in the neglected side. Because it is often the case that individuals with acquired peripheral dyslexias provide mainly lexical responses, the word list was created so that an omission, substitution, or addition of letters on the left or on the right of each of the target words would create existing words.

As will be reported in the Results, most of our participants' neglect error responses (91%) were indeed existing words. Therefore, each of the analyses was made out of the set of words that could be created by a neglect error of the relevant type. For example, for the participants with left neglect, the word Š*oReK* has lexical potentials for omission, substitution, and addition (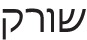 → 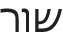, 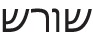, 
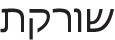
; *šorek* → š*or/šoreš/šoreket*)—namely, each of these error types could create an existing word; the word *tarnegolim* had lexical potential for omission and substitution (
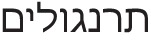
 → 
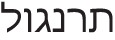
, 
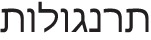
; *tarnegolim* → *tarnegol*/*tarnegolot*), but not for addition—namely, no existing word could result from an addition of a letter to the left of this target word; the word *nafsik* only has the lexical potential for substitution (
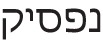
 → 
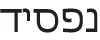
; *nafsik* → *nafsid*). Thus, each analysis was made out of the words that had the relevant lexical potential: omissions were calculated only out of the total number of words that allowed for an omission that would create an existing word, and the same for substitutions and additions. Therefore, in the analysis of the total number of words with a lexical potential for omission, words like *shorek* and *tarnegolim* were included, but not the word *nafsik*.

The potential word sets also took into account the neglect point of each participant (e.g., for participants who tended to only neglect the final letter in 4–5 letter words, the potential sets were created accordingly, for words that differ in the final letter only). Potential words that produced infrequently used words were not included (see Section 3.7.4 for the relative frequency of the target word and the lexical error responses).

#### 2.3.2. Real morphological components vs. potentially-morphological components

A component that can be used as a morpheme can be a real morpheme, namely, function as part of the affix in the target word (like –er in *dancer* in English), or can be potentially morphological, namely, include the letters and be placed in a position in the word that could function as an affix in some words, but not be part of the affix in the target word (like –er in *corner*). To determine whether a component that can be used as a morpheme has a real morphological role or a potentially morphological role in the specific target word, a list of the relevant words was presented to 10 linguists and psycholinguists who are native speakers of Hebrew. Only words for which the agreement rate with respect to the status of the affix was higher than 70% were included in the analysis comparing real and potential morphological role.

### 2.4. Statistical analyses

A comparison between conditions for each participant individually was performed using chi-squared (χ^2^) tests or Fisher tests, according to the number of items compared. In all of the tables in the paper, the chi-square values are reported using the χ^2^ and *p*-values, and the Fisher's exact probability test is presented with a *p*-value. A comparison of the error types at the group level was performed using *t*-test, reported with a *t*-value. The logistic regression coefficients (*B*-values) are reported, and the binominal tests are presented using z statistics. All tests were conducted with α = 0.05. A non-significant difference was defined as a trend when 0.05 < *p* ≤ 0.1.

## 3. Results

The same analyses were done for the 6 participants with left neglexia and for the participant with right neglexia. We will first present the analyses and findings from the participants with left-sided neglexia in Sections 3.1–3.7, and then in Section 3.8, the findings from the participant with right-sided neglexia will be presented.

### 3.1. Reading accuracy and error types

The participants with left-sided neglexia had between 15% and 57% left-sided neglect errors when reading the word lists, with a group mean of 26% errors (Table 3). Almost all the errors the participants made were neglect errors, namely, errors of omission, substitution, or addition of letters on the left of the word, and none of the participants had more than two non-neglect errors– errors that were not confined to the left of the word. Such non-neglect errors amounted to only 1.1% of the total number of words the participants read, supporting the participants' diagnosis of left neglexia. The eight non-neglect errors were excluded from further analyses.

**Table 3:** **Left-sided neglect errors: number and rate of left-neglect errors compared with other non-left errors out of all words presented, and the rate of lexical responses out of the neglect responses of each participant**.

	**Neglect errors of total target words**		**Non-neglect errors of total target words**		
**Participant**	**Neglect Errors/Total**	**% Neglect errors**	**Non-neglect errors/Total**	**% Non-neglect errors**	**% Lexical responses of neglect responses**
B	30/116	26	0/116	0	93
H	27/88	31	0/88	0	100
Z	62/108	57	2/108	2	77
C	29/126	23	2/126	2	100
T	24/163	15	2/163	1	92
K	23/138	17	2/138	1	100
**Total**	195/739	**26**	8/739	**1**	**91**

Most of the neglect error responses of the participants with left-sided neglexia (91%) were existing words. The neglect errors yielded significantly more lexical than non-lexical (non-word) responses both at the individual level (χ^2^ ≥ 37.29, *p* ≤ 0.001) and at the group level (*z* = −11.39, *p* < 0.0001). Only one participant (Z.), who had the highest rate of neglect errors (57% of the words he read), produced more than two non-lexical responses. As a result, we calculated the rate of each type of error out of the target words with a lexical potential of the relevant type. For example, left sided letter omissions were calculated out of the number of words the participant read for which a left letter omission could create an existing word (see Methods Section).

The neglect errors the participants made included letter omissions (e.g., 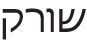 → 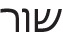; ŠoRQ → ŠOR; š*orek* → š*or*), letter substitutions (e.g., 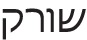 → 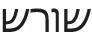; ŠoRQ → ŠoRŠ; š*orek* → š*oreš*), and letter additions (e.g., 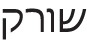 → 
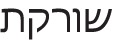
; ŠoRQ → ŠoRQt; š*orek* → š*oreket*). Although the participants made a larger number of substitution errors (see Table 4), this is a result of the number of words in the list that allowed for lexical substitution errors compared with lexical omissions or additions. When the errors of the various types are calculated as rates out of the number of words in which such an error would create an existing word, the rate of omissions, substitutions, and additions becomes similar (Table 4). There were similar rates of the various neglect error types at the group level [*t*_(5)_ ≤ 1.04, *p* ≥ 0.53]. Similarly, at the individual level, except for T. and C., the analysis of the rates of the three types of neglect errors yielded no significant differences between the different error types (*p* ≥ 0.08). T. had significantly more substitutions than omissions (*p* = 0.008) and made only one omission error. C. had significantly more omissions than substitutions (χ^2^ = 4.48, *p* = 0.03). Table 4 presents the distribution of neglect errors of the three types out of the lexical potential for each type.

**Table 4:** **The distribution of neglect errors out of the words with a lexical potential for error of each type**.

**Participant**	**Omission**		**Substitution**		**Addition**	
	**Errors/Total**	**% Errors**	**Errors/Total**	**% Errors**	**Errors/Total**	**% Errors**
B.	7/68	10	10/109	9	8/41	20
H.	8/49	16	9/80	11	3/27	11
Z.	12/46	26	16/76	21	6/31	19
C.	12/63	19	10/120	8	6/47	13
T.	1/77	1	15/145	10	2/56	4
K.	2/63	3	12/125	10	3/49	6
**Total**	42/366	**11**	72/655	**11**	28/251	**11**

### 3.2. The effect of morphology on reading: root vs. affix

The first analysis of the role of morphology on reading in neglexia assessed the rate of neglect errors as a function of the morphological status of the left side of the word. Throughout the article, we will use the term “affix” to refer to non-root letters that are part of the nominal or verbal derivational pattern morpheme, or part of an inflectional morpheme. These could occur as an infix, suffix, prefix, or a combination thereof. For the analysis of left-sided neglexia we will use the term “affix” for non-root morphemes that appear in the left side of the word.

We compared the rate of neglect errors (letter omission, substitution, and addition) in words that end (left side) in a root letter (including real and potential roots, see 3.7.2) with words that end in an affix (real or potential, Methods section). As shown in Table 5, all the participants neglected more letters belonging to affixes than root letters. This difference was significant at the group level and for four of the individual participants.

**Table 5:** **Neglect of a root letter in words ending with a root letter and neglect of an affix letter in words ending with an affix**.

**Participant**	**Ending with a root letter**		**Ending with an affix**		**Comparison**
	**Errors/Total**	**% Errors**	**Errors/Total**	**% Errors**	
B.	2/39	5	16/53	30	***p* = 0.002**
H.	5/30	17	14/40	35	χ^2^ = 2.91, *p* = 0.09
Z.	7/34	21	24/39	62	**χ^2^ = 12.47, *p* = 0.0004**
C.	2/44	5	16/51	31	***p* = 0.001**
T.	6/54	11	11/63	17	χ^2^ = 0.94, *p* = 0.33
K.	3/49	6	12/51	24	***p* = 0.01**
**Total**	25/250	**10**	93/297	**31**	***t*_(5)_ = 4.35, *p* = 0.002**

*In this table and in all of the following tables, the boldface in the comparison column marks a significant difference*.

To rule out a confound of length effect that may have modulated the morphological effect (words ending with a root letter had 3–5 letters, *M* = 4.1 letters, whereas the words ending with an affix had 4–8 letters, *M* = 5.2 letters), we compared neglect errors only in 4- and 5-letter words ending with a root or with an affix. In this analysis too, there were significantly more neglect errors in words ending with an affix: for 4-letter words, there were 13% errors in words ending in a root letter and 29% errors in words ending in an affix. For 5-letter words, the rates were 12 and 24%, respectively. In 4- and 5- letter words analyzed together, the left letter was neglected significantly more often when it belonged to an affix (27%) than when it belonged to the root (13%), *t*_(5)_ = 2.09, *p* = 0.04. Thus, the morphological role effect in left-sided neglexia is a real effect and cannot be explained by the length effect.

In conclusion, the reading of participants with neglexia was found to be affected by the morphological role of the left side of the target word: significantly more neglect errors occurred when the left side of the word was part of an affix than when it was part of the root.

### 3.3. Does the morphological effect result from morphological decomposition of the target word?

A question that arises from these findings is whether letters that are part of the affix are just recognized as letters that can, in general, have a morphological role in some words, or whether, for each word, a morphological analysis of the target word is made that identifies the root and template/inflection, and then the letter is treated as an affix letter when it can be part of the affix in the specific target word, at least according to a structural analysis of the word.

A way to determine between these possibilities comes from the fact that in Hebrew all the letters that can serve as part of an affix can also be part of the root. We used this property of Hebrew to compare between two possible explanations: one according to which there is no decomposition but only a list of affix letters, and another explanation according to which the target word undergoes morphological decomposition. We did so by comparing the neglect of the same letters in two roles. Specifically, we compared letters that can take an affix role in some words, when they function as an affix and when they function as the third letter of the root. To do this, we compared neglect error rates in words ending with the letters *m* (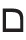) and *n* (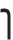) when they function as an affix (e.g., in the word 
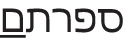
, SPRtm, *safartem, count*-past-2nd-mas-pl, where the *m* serves as part of the inflection) and when they function as a root letter (e.g., in the word 
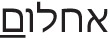
, aXLoM, *axlom, dream*-future-1st-sg, where the *m* serves as the third root letter). Lexical knowledge is not required to identify the letter in the two words as part of the affix or as part of the root: the structure of the words and its derivational templates and inflections indicates whether it is (structurally) a root or an affix letter.

As shown in Table 6, this comparison indicated that the participants with neglexia neglected the exact same letters in exactly the same linear position significantly more often when, taking into account the structure of the whole word, these letters functioned structurally as affixes in the target words than when they were part of the root. All the participants showed this pattern, which was significant for B. and Z.

**Table 6:** **Neglect errors (omissions and substitutions) in the left letters *m* and *n* when they appear as part of the affix and as part of the root**.

**Participant**	**Ending with a root letter**		**Ending with an affix letter**		**Comparison**
	**Errors/Total**	**% Errors**	**Errors/Total**	**% Errors**	
B.	0/5	0	8/15	53	***p* = 0.05**
H.	0/4	0	2/11	18	*p* = 0.52
Z.	0/5	0	6/11	55	***p* = 0.06**
C.	0/5	0	1/12	8	*p* = 0.71
T.	0/6	0	1/17	6	*p* = 0.74
K.	0/6	0	3/14	21	*p* = 0.32
**Total**	0/31	**0**	21/80	**26**	***t* = 16.6, *p* < 0.0001**

Thus, this comparison indicates that neglect is influenced by the morphological role of the letter in the target word: a root letter or an affix letter, and not by a list of letters that could function as an affix and are thus deleted regardless of their role in the target word. It suggests that an analysis of the structure of the whole word is done, probably on the basis of information about templates and affixes in Hebrew and the search for three consonant letters to serve as a root. This, in turn, indicates that an early morphological analysis of the whole word occurs prior to the stage at which letters are neglected.

### 3.4. The effect of morphology on different types of neglect errors: no omissions of root letters

An analysis of the different types of neglect errors in words ending with a root letter and in words ending with an affix, summarized in Table 7, showed that the morphological status affected different neglect errors differently. In target words ending with a root letter, there were significantly fewer omissions than substitutions and additions. For words ending with an affix, no significant difference was found between the rates of the different types of neglect errors.

**Table 7:** **The rate of different types of neglect errors in words ending with a root letter vs. words ending with an affix**.

	**Ending with a root letter**		**Ending with an affix letter**		**Comparison**
	**Errors/Total**	**% Errors**	**Errors/Total**	**% Errors**	
**ERROR TYPE**					
Omission	1/84	**1**	37/211	**18**	***B* = −2.87, *p* = 0.005**
Substitution	19/232	**8**	42/273	**15**	***B* = −0.71, *p* = 0.02**
Addition	18/158	**11**	2/18	**11**	*B* = 0.03, *p* = 0.97
**COMPARISONS BETWEEN ERROR TYPES**					
Omissions-substitutions	***B* = −2.00, *p* = 0.05**		*B* = 0.16, *p* = 0.53		
Omissions-additions	***B* = −2.37, *p* = 0.02**		*B* = 0.01, *p* = 0.99		
Substitutions-additions	*B* = −0.37, *p* = 0.29		*B* = 0.38, *p* = 0.63		

*The analysis summarized in this table includes only words with the relevant lexical potential for each type of error, only lexical neglect errors, and excluding errors that occurred after the first or second letter*.

Furthermore, the morphological role affected omissions and substitutions, but not additions: omissions and substitutions occurred more often in words ending with an affix than in words ending with a root letter. For addition errors, no significant difference was found between the two types of words.

The most striking difference between root and affix letters was thus found in the rate of omissions. Why are omissions so sensitive to the morphological status of the letters in the neglected side? In Hebrew, most words are constructed from 3-letter roots and affixes, the root carries most of the meaning of the word, and is probably the unit stored in the orthographic input lexicon. We believe that the sensitivity to morphology results from this fact. The results suggest that orthographic-visual analysis is directed by a search for three letters of the root, and the orthographic-visual analyzer refuses, as it were, to stop before it identifies three root letters. This creates the situation in which root letters on the neglected side are almost never omitted. In the reading of all the words ending with a root letter with a potential for omission, across all participants, only a single omission of a root letter was made. It seems that the visual analyzer does not stop shifting attention to the left until three consonant letters that could form the root have been identified.

This pattern also has a direct effect on whether or not the neglect response keeps the length (number of letters) of the target word. In a general analysis across all word types, none of the participants preserved word length, only 33% of the responses preserved the length of the target word. There were more neglect errors that did not preserve word length than neglect errors that preserved word length (a Binomial analysis that pulled all the responses of the participants together, *z* = −4.61, *p* < 0.0001). This is related to the finding that, as shown in Table 4, letter omissions and additions, which changed the length of the word, also occurred, and not only substitutions that preserved word length. Once the preservation of word length is analyzed (see the bottom of Table 7), with a separate analysis of words ending with a root letter and with an affix, one can see that there were almost no responses that shortened the word length when the target word ended with a root letter, whereas for words ending with an affix, no significant difference was found between the rates of neglect errors shortening, elongating, or keeping the original word length.

### 3.5. Interim summary: the effect of morphology on reading in neglexia

The morphological role of the neglected side of the word has a crucial effect on reading in neglexia: letters on the left side of the word are neglected more often when they function as an affix in the target word than when they function as root letters. This effect is a result of the morphological analysis of the target word and identification of the role of each letter in the target word, as the same letters can sometimes be treated as affixes, and be neglected, or as root letters, and be retained, according to the morphological structure of the target word. The morphological structure is analyzed as a whole, based on knowledge of the morphological structure of Hebrew words, and hence, of possible structures in which the root letters are inserted: the derivational and inflectional templates. The morphological role of the letter mainly affects omission and substitution errors. This indicates that the orthographic-visual analyzer is actively searching for the three root letters. Until these root letters have been detected, attention shifting continues, and these letters are not omitted. When the three root letters are identified, there is no longer difference between words ending with an affix and words ending with a root, and letter additions occur in both word types to a similar extent.

### 3.6. Perceptual effects in reading in neglexia are modulated by morphological structure

The finding that the morphological structure of the word affects reading in neglexia, which is a pre-lexical impairment, already points to a pre-lexical morphological decomposition. To further examine the locus of morphological decomposition, we examined the effect of perceptual factors, length effect, and final letter-form effect, on the reading of participants with neglexia.

The rationale was that if these perceptual effects differentially affect words that end in a root letter and words that end in an affix, morphological decomposition occurs very early, at the stage in which these perceptual effects apply. We evaluated the existence of these effects for words of all morphological types together, and then moved to assess whether these perceptual effects affect roots and affixes to the same degree.

#### 3.6.1. Length effect is modulated by morphological status

To evaluate the effect of the number of letters in the word on reading, we compared the error rates in words of different lengths: 3 letters, 4 letters, 5 letters, and 6–8 letters. In this analysis, all types of neglect errors were included in the calculation of number of errors, including non-lexical responses. As shown in Table 8, four participants showed significantly more errors in longer words, an effect that was significant at the group level too, as indicated by pairwise comparisons as well as a significant linear contrast [*F*_(1, 5)_ = 15.25, *p* = 0.01], showing a linear increase in the error rates with the increase in word length.

**Table 8:** **Neglect error rates in words of different lengths (words ending in a root letter and words ending in an affix together)**.

**Participant**	**3 Letters**	**4 Letters**	**5 Letters**	**6+ Letters**
B.	17%^6^	23%	21%	45%^3^
H.	27%	24%	38%	37%
Z.	41%^6^	59%	61%	75%^3^
C.	24%	28%	18%	27%
T.	0%^4, 5, 6^	17%^3^	14%^3^	32%^3^
K.	0%^4, 5, 6^	15%^3, 6^	19%^3, 6^	43%^3, 4, 5^
**Total**	17%^4, 6^	27%^3, 6^	24%^6^	44%^3, 4, 5^

*The numbers in superscript indicate the lengths that were found to be significantly different. For example, for participant B., a significant difference in the error rates was found between 3 letter words and words with 6–8 letters*.

Importantly, when the calculation of length effect was done separately for words ending with a root letter and words ending with an affix, a different picture emerged. For words ending with an affix, there were more neglect errors in 6–8 letter words than in 5-letter words, whereas for words ending in a root letter, there was no difference in error rates between words of different lengths. In order to assess the effects of word length and word category on subjects' error rates, logistic regression with two-way interaction (Word Category X Length) was calculated. This interaction was significant (WALD = 6.31, df = 2, *p* = 0.04), meaning that the word length affected subjects' error rates differentially according to word category. Namely, once the word ended with a letter that was part of the root, the error rate did not increase when the word became longer. Further analysis revealed that this interaction was due to the difference in error rates between 6 and 8 letter words and 5-letter words for words ending with an affix (WALD = 5.14, df = 1, *p* = 0.02).

Relatedly, the presence of a prefix (on the right-hand side of the word) in words ending with a root letter did not raise the neglect error rate in comparison with words without a prefix (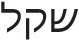
—
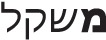
, **m**ŠQL—ŠQL, *miškal* vs. š*ekel*), both at the individual level (*p* ≥ 0.13) and at the group level (*t*_(5)_ = 1.3, *p* = 0.12). This finding indicates that the prefix letter is identified as such and is not counted as a root letter.

In summary, words ending with a root letter did not show a length effect, whereas words ending with an affix did show a length effect for 5-letter and 6–8 letter words.

#### 3.6.2. Final letter form effect is modulated by morphological status

Hebrew has five letters that change their form according to their position in the word. When they appear in the final (leftmost) position in the word, they bear a different form than when they appear in any other position. These letters have the form 
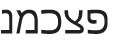
 in the beginning or middle of the word, and 
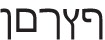
 in final position (Friedmann and Gvion, 2005). To assess the effect of the letter-form (final-non final) on reading, we compared words ending with a final-form letter with words ending with a letter that does not change its form at the end of the word (from here on “non-final letters”).

All of the participants except B. had more neglect errors in words ending with a non-final letter than in words ending with a final letter. This difference was significant for H., Z., and C. (*p* ≤ 0.03). At the group level, there were more neglect errors in words ending with a non-final letter than in words ending with a final letter (*t*_(5)_ = 2.06, *p* = 0.04)^4^.

Similarly to the length effect, the effect of final letter forms on neglect errors was modulated by morphology. Whereas when all the target words are analyzed together, significantly more neglect errors were made in words ending with a non-final letter than in words ending with a final letter, the analysis by morphological status showed that the final letter effect was found in words ending with an affix but not in words ending with a root letter. For words ending with a root letter, no significant difference was found between words ending with final and non-final letters, both at the individual level (*p* ≥ 0.35) and at the group level (*t*_(5)_ = 0.97, *p* = 0.18). In contrast, for words ending with an affix, the group (without B who showed a reverse trend) made significantly more neglect errors in words ending with a non-final letter than in words ending with a final letter, *t*_(4)_ = 2.28, *p* = 0.04. This effect applied for each of the individual participants, except B., but was significant only for C. (*p* = 0.05).

#### 3.6.3. Interim summary: morphological structure affects the manifestation of perceptual effects

Whereas in the calculation of all test words, length and final letter effects were found, these perceptual factors did not affect the reading of words ending with a root letter, only words ending with an affix. Different patterns were also found with respect to neglect errors of different types (omission, substitution, and addition) for the words ending in a root letter vs. words ending in an affix, indicating the greater resilience of words ending with a root letter in comparison to words ending with an affix. The finding that these perceptual effects show differential behavior for words ending in root and affix letters indicates that morphological decomposition occurs very early, at the orthographic-visual perception stage in which the perceptual effects apply.

### 3.7. Does morphological decomposition occur before access to the lexicon and to meaning?

If morphological decomposition is indeed implemented in an early, pre-lexical stage, before the access to the lexicon and to meaning, and without feedback from the lexical stages, we would not expect semantic and lexical variables to affect the reading of the participants with neglexia. We thus examined whether various lexical and semantic factors affect their reading and the manifestation of the morphological effects on their neglect errors. Absence of such effects would support pre-lexical morphological decomposition.

#### 3.7.1. Words for which a structural non-lexical morphological decomposition creates a lexically incorrect analysis

One way to examine whether the morphological decomposition occurs at a stage at which lexical factors already play, or whether it is guided by purely structural characteristics of the target word, is by examining the reading of words that “trick” or mislead a pre-lexical structural analysis. We used words ending with an affix letter that an early structural morphological decomposition, ignorant of lexical knowledge, would analyze as a root letter. For this analysis we used words that have a defective root of only two letters and a consonantal affix, which could be taken by structural non-lexical analysis to be the third consonant. The rationale was the following: to know that in this specific word there are only two root letters and the final letter is an affix letter, one needs to access the lexicon. Otherwise, a preliminary structural morphological decomposition would take the final consonant to be the third root consonant. Thus, such defective roots offer a way to find out whether the morphological analysis and its effect on neglect errors take into account lexical considerations. If these words behave like words ending with a root, and include fewer omissions than words ending with an affix, this will indicate that the morphological analysis in this stage is structural, and is not guided by lexical considerations. Namely, that the morphological analysis that affects neglect errors is pre-lexical.

For example, the word 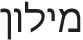 (MiLon, *milon*, dictionary) is derived from the word 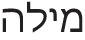 (MiLh, *mila*, word) plus the derivational affix 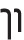
- (-on). However, this knowledge, and the relation between *word* and *dictionary*, only exist in lexical and semantic stages. Structurally, because the base only has two consonant letters, this word could be analyzed as a word with a 3-consonant root, if the affixal -n is taken to be the third root consonant. To allow for a comparison between words with defective and 3-letter roots, we used words with similar frequencies (*M* = 4.3, *SD* = 1.04, for the defective root words, and *M* = 4.2, *SD* = 1.16, for the other words we tested, which included three letter roots).

The results were that the participants with neglexia treated these words as if they ended in a root letter, namely, they did not use the information in the lexicon about this word, which would have caused them to treat it as ending with an affix. Each of the participants made fewer neglect errors in these “unclear” words than in words with three root letters clearly ending with an affix, and this difference was significant for B. and C. (*p* ≤ 0.04). Furthermore, these “tricky” words behaved like the words that end with a root letter: all the participants showed similar neglect error rates for the “tricky” words and for words ending with a root letter, *p* ≥ 0.25 (and B. even showed marginally significantly fewer errors in the tricky words compared with the root-ending words). And so did all of them as a group, *t*_(5)_ = 1.04, *p* = 0.17.

Therefore, we can conclude that morphological decomposition at this stage is structural rather than lexical-semantic, and treats words with only two root letters and a final consonant affix letter like three-consonant root words, and considers the left letter to be a root, rather than an affix letter, and hence does not neglect it. These results also indicate that the morphological effect is a result of morphological analysis of the whole target word rather than a different, simple, treatment of letters that belong to a list of “morphological letters.” These results thus indicate that the morphological analysis is structural and can occur without information from the lexical level.

#### 3.7.2. Does the lexicality of the root affect decomposition?

Another way of examining whether morphological decomposition occurs before the lexicon and whether it is influenced by the lexicon and semantics is by examining whether the decomposition occurs only when a productive root (i.e., a root that acts as a root in additional semantically-related words) is identified or whether it occurs in every case in which the word structure enables the identification of three consonant letters that can serve as root letters. To examine this, we compared the neglect error rate in words in which the left letter is part of a real productive root with the error rate in words in which the left letter is part of a consonant sequence that is structurally the root but is not a real productive root.

We defined a sequence of consonants a productive root if the target word was a 3-consonantal verb, or if there was a 3-consonantal verb or an action noun derived from the same root and semantically related to the target word. E.g., the word 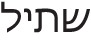 (ŠTiL, š*til, seedling*) includes a real productive root, because its root, STL, serves in the verb 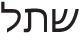 (ŠTL, š*atal, planted*), which is semantically related to it.

No significant difference was found between the neglect error rates in words ending with a productive root letter and in words ending with a potential root letter, at the individual level (*p* ≥ 0.24) and at the group level (*t*_(5)_ = 0.24, *p* = 0.41). Thus, words in which three consonants can structurally serve as a root, even if they are not real productive roots, are morphologically decomposed just like words with a meaningful productive root.

#### 3.7.3. Does it matter if the affix letter really functions as an affix in the target word?

A similar comparison was conducted for affixes. We analyzed words ending with an affix letter, comparing words ending with a real affix and words ending with a potential affix. A word was defined as ending with a real affix if it included a real 3-letter root or stem that was joined to the affix, and the root/stem was semantically related to the affixed word (e.g., *dancer* in English). A word was defined as ending with a potential affix if it included three letters with the potential to act as a root that were joined to letters with the potential to be an affix, but the root/stem was not semantically related to the affixed word (e.g., *corner* in English).

In this comparison too, no significant difference was found between words ending with a real affix (96/278) and words ending with a potential affix (4/19), at the individual level (*p* ≥ 0.22) and at the group level (*t*_(5)_ = 1.71, *p* = 0.07).

These comparisons, at the root and at the affix levels, provide evidence that there is no lexical-semantic effect on the morphological analysis that affects neglect errors, and that this preliminary morphological decomposition does not take the existence of a real root or the semantic relationship between the decomposed word and the target word into account.

#### 3.7.4. No clear frequency effect

Another way to evaluate lexical effects on reading was by assessing whether word frequency, which is clearly a lexical factor, affected reading accuracy and neglect errors. We evaluated the relative frequency of the target and response words, as well as the correlation between the target word frequency and the success in reading it.

To examine the relative frequency of the target words and the erroneous responses the participants provided, we presented 30 skilled readers, native speakers of Hebrew, with pairs of words that included the target word and the erroneous response word. The judges were asked to mark the more frequently used word of the two or to mark both of them if they felt that the words had similar frequency. To include only target-response pairs for which there was a clear frequency difference, the target word was defined as more frequent if the ratio [number of judges who chose the target as more frequent/(2^*^ number of judges who chose the response as more frequent + number of judges who judged the words as similar)] was at least 1.5. The response word was defined as more frequent in the same way, namely if [response/(2^*^target + similar)] was at least 1.5.

To examine the relation between frequency and the participants' performance, the frequencies of the target words were collected through the judgments of 30 native Hebrew speakers. In this judgment, the judges rated the frequency of the word on a 7-point scale from “very rare” to “very frequent.”

In the analysis of the relative frequency of the target and response, the participants' performance was characterized by mixed trends. Two of the participants, H. and Z., had a significantly higher percentage of erroneous responses that were more frequent than the target words *(p* ≤ 0.04), three participants showed no significant difference between the two types of responses, and one participant, T., had a significantly higher percentage of erroneous responses that were less frequent than the target words (*p* = 0.02).

To examine the effect of frequency on accuracy, we ran logistic regression with error rates as dependent and word frequency as independent variables. K's error rate was found be dependent on word frequency (*B* = −0.49, *p* = 0.03). B's error rate was marginally depended on word frequency (*B* = −0.39, *p* = 0.06). The other four participants did not show dependence between error rate and word frequency (−0.20 ≤ *B* ≤ 0.06, *p* ≥ 0.33).

#### 3.7.5. No semantic effects

Another analysis we used to examine whether lexical-semantic factors affect neglect errors focused on the semantic relation between the response and the target word.

##### 3.7.5.1. Semantically related and unrelated responses

We compared neglect errors that result in words semantically related to the target word (e.g., 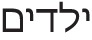 → 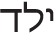, ILDim → ILD, *boys* → *boy*) and neglect errors that result in words with no semantic relation to the target word (e.g., 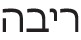 → 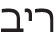, RIBH → RIB, *jam* → *quarrel*). The analyses were performed on words ending with an affix letter (real or potentially morphological affix).

No significant difference was found between neglect errors that created words semantically related to the target words and neglect errors which were not semantically related to the target words, at the individual level and at the group level [*t*_(5)_ = 1.7, *p* = 0.07]. Namely, there was no effect of the semantics of the target word on the erroneous response produced.

##### 3.7.5.2. No preservation of morpho-lexical features

We also examined whether the neglect errors preserved morpho-lexical features of the target word, such as the lexical category and gender. Preservation of these features can provide evidence that higher processing occurs prior to morphological decomposition, because to know the lexical category and gender of a written word, the reader has to access the syntactic lexicon (Friedmann and Biran, 2003; Biran and Friedmann, 2012). Preservation of morphosyntactic properties of the target word would thus provide evidence that such access to lexical stages has occurred prior to the morphological decomposition, and hence, would indicate that the morphological decomposition is post-lexical.

The analysis in this section only included words for which neglect errors of any type had both the potential for creating a word that preserves the relevant feature and a word that does not preserve this feature (e.g., one of the words in the analysis of lexical category preservation was the noun 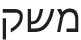, MŠQ, which could be read with a neglect error as another noun, 
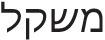
, mŠQL or as a verb, 
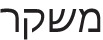
 mŠQR). We then compared the rate of errors that preserved the relevant feature and errors that did not^5^.

No significant difference was found between neglect errors that preserved the lexical category (noun, verb, adjective) and neglect errors that did not preserve the lexical category, at the individual level (χ^2^ ≤ 2.89, ≥ 0.13) and at the group level (*z* = 0.58, *p* = 0.72).

As for the gender feature, in Hebrew there are two grammatical genders, masculine and feminine, both for animate and for inanimate nouns. Adjectives and verbs also inflect for one of the two genders. We tested whether neglect responses preserved the gender or the gender inflection of nouns, adjectives, and verbs. The results indicated that there was no tendency to preserve the gender of the target word in the response, and in fact four of the participants even had a smaller percentage of neglect errors that preserved the gender feature than neglect errors that did not preserve this feature, and for C. this difference was significant (χ^2^ = 5.33, *p* = 0.02). For K. no difference was found between the two types of neglect errors. Thus, these findings indicate that there is no tendency to preserve lexical categories or gender inflection in neglect errors.

##### 3.7.5.3. Derivational vs. inflectional errors

Some studies of Hebrew normal reading suggested that some types of morphemes are decomposed but others are not (Deutsch et al., 1998; Frost et al., 2000b, for example, demonstrated differences between verbal and nominal templates). We examined this issue by comparing neglect errors that reflect inflection processes and neglect errors that reflect derivation processes.

In an analysis of the errors that took into account for each target word the lexical potential for derivational and inflectional errors, no significant difference was found between derivational omissions and inflectional omissions either at the individual level (*p* ≥ 0.06) or at the group level [*t*_(5)_ = −0.36, *p* = 0.63]. In the analysis of substitution errors, also no significant difference was found between derivational substitutions and inflectional substitutions both at the group level [*t*_(5)_ = 0.45, *p* = 0.33] and at the individual level, at which none of the participants showed a significant difference between the two types of substitutions (*p* ≥ 0.45), except for B. (*p* = 0.04). Similarly, in the analysis of addition errors, no significant difference was found between derivational additions and inflectional additions at the group level [*t*_(5)_ = −0.13, *p* = 0.55], and at the individual level, at which none of the participants showed a significant difference between the two types of additions (*p* ≥ 0.36), except for C. (*p* = 0.04). Thus, the distinction between derivational and inflectional morphology did not have an effect on the participants' performance, and it seems that both types of morphemes are decomposed at the pre-lexical morphological decomposition stage.

#### 3.7.6. Interim summary: morphological decomposition is structural and prelexical

The findings in this section indicate that lexical and semantic factors do *not* affect the neglect pattern of the participants with neglexia. These results indicate that neglect errors occur before written words undergo lexical and semantic processing, and without feedback from these stages.

Indeed, we know that the lexicon affects reading in neglexia in general—a word like *artichoke* is likely to be read correctly, because no other word exists that results from an omission or substitution of the left letter of the word, and hence, access to the lexicon with the partial information about the letters would activate a single word—artichoke, and the word would be read correctly, unlike the word *rice*, for example, which could be read as *nice, ice, price* etc.

However, such lexical considerations could not be the source of the pattern of morphological structure effect that we see here: the words that end with a root letter and the words that end with an affix letter showed different error patterns even though both were selected such that neglect errors would create in each of them existing words. Furthermore, we saw the morphological effects even in pseudo-roots and in defective 2-letter roots that were treated by the structural morphological analysis as 3 letter roots, namely, where there was no lexical support from the constituents.

Therefore, we suggest that the morphological effect results from an earlier stage, of a non-lexical non-semantic preliminary morphological decomposition, that is guided by the morphological structure of the target word and affects the attention shift itself. A relevant metaphor would be a city in which all streets have 5-letter flower names. When one sees a street sign in this city, which is partly covered by a traffic light pole, and hence only sees four letters, he will move his head to see the fifth letter. This is parallel to the shift of attention to access the third letter of the root. If this sign is too far and hence looks blurry, then the lexicon can be helpful if only some of the letters are more easily identified: if the reader, after moving his head sees “?aisy” the lexicon would help and activate the word “daisy.”

### 3.8. The effect of morphology on reading in right-sided neglexia

The reading of R., the participant with right-sided neglexia, was also significantly affected by the morphological status of the neglected side: R. made significantly more neglect errors in words in which the beginning (the right side) was an affix^6^ (15/24, 63%) than in words that began with a root letter (7/22, 32%; χ^2^ = 4.33, *p* = 0.04).

Similarly to the participants with left-sided neglexia, R. made significantly fewer omissions in words beginning with a root letter (5/21, 24%) than in words beginning with an affix (12/22, 55%; χ^2^ = 4.25, *p* = 0.04). Moreover, and also similarly to the participants with left-sided neglexia, whereas for words beginning with an affix, significantly more omissions were made than substitution errors (*p* = 0.001), for words beginning with a root letter, no significant difference was found between the rates of various types of neglect errors (*p* ≥ 0.21).

Similarly to the findings on left-sided neglexia, R.'s reading was not affected by lexical and semantic factors, suggesting that morphological decomposition occurs prior to access to the lexicon and to meaning also in right-sided neglexia.

#### 3.8.1. Real vs. potential root

No significant difference was found in the rate of neglect errors between words beginning with a real root letter (6/17) and words beginning with a potential root letter (1/5; *p* = 0.48).

#### 3.8.2. Frequency

No significant correlation was found between the target words' frequency and R.'s success in reading them (*B* = −0.24, *p* = 0.27). There was no tendency to produce an error that is more frequent than the target word. In fact, R. made significantly more errors that were less frequent than the target word (38%) than errors than were more frequent than the target (7%), *p* = 0.005.

#### 3.8.3. Semantically related vs. semantically unrelated

No significant difference was found between affix neglect errors that created a response semantically related to the target word (9/29) and affix neglect errors that were semantically unrelated to the target word (5/23; χ^2^ = 0.56, *p* = 0.45).

#### 3.8.4. Derivational vs. inflectional errors

No significant difference was found between the rate of derivational neglect errors (7/23) and inflectional neglect errors (2/9; *p* = 0.64).

#### 3.8.5. Preservation of morpho-lexical features (lexical category and tense)

There was no significant difference between neglect errors that preserved the lexical category of the target word and neglect errors that did not preserve this feature (χ^2^ = 2.89, *p* = 0.13). Additionally, for right-sided neglexia, we examined the preservation of a morphological feature that appears in the right side of the word—the tense inflection. R. made significantly more neglect errors that changed the tense inflection (8/10) than neglect errors that preserved the tense inflection of the target word (2/10; *p* = 0.01).

In summary, the performance of the participant with right-sided neglexia was consistent with the findings from left-sided neglexia in relation to the effect of the morphological structure of the word on reading performance and to the characteristics of this effect: words beginning with an affix letter were more susceptible to neglect errors than words beginning with a root letter, and the morphological effect on reading was not affected by lexical or semantic factors, a finding that also locates the morphological effect on reading in right-sided neglexia as occurring during visual-orthographic analysis, and pre-lexically.

## 4. Discussion

This study explored morphological decomposition in reading, its nature and where in the process of word reading it occurs. These questions were explored through the analysis of neglect errors in the reading of seven Hebrew-readers with neglexia and the effect of the morphological structure of the target words on their reading. The main findings of this study are:

- The morphological structure of the target words affected the reading of the participants with left-sided neglexia and the participant with right-sided neglexia: more neglect errors occurred when the neglected side of the word was an affix than when it was part of the root^7^.
- This morphological effect was especially robust in omissions: root letters were almost never omitted from the neglected side, a finding we ascribed to the effect of the search for three root letters on attention shifting in neglexia.
- Letters that can serve both as affixes and as root letters were neglected when they were structurally an affix in the target word, but were not omitted when the structure of the word determined that they could function as root letters.
- Perceptual effects of word length and letter form affect words ending with an affix but not words ending with a root. The finding that the stage at which perceptual factors play a role is already subject to morphological modulation indicates that the morphological decomposition occurs early, at the stage in which the perceptual effects take place.
- This morphological decomposition is structural-orthographic and is not affected by lexical considerations. It is not affected by the lexical status of the root (whether it is a real productive root or just a structurally possible one), by the affixal status of the affix in the target word (whether it is a real affix in the word or only a potential one), or by whether a consonant letter that appears after two consonantal root letters is lexically an affix. The absence of lexical support is also demonstrated by the findings that the reading of the participants did not show a clear frequency effect.
- Semantic factors do not affect neglect errors. Neglect responses do not necessarily have a semantic relation to the target word, and do not preserve the morpho-lexical features of the target word such a gender, tense, or lexical category. This further supports the conclusion that early morphological decomposition occurs prior to lexical and semantic processing, and can occur without any feedback from these stages.

Taken together, these findings indicate that a preliminary structural morphological decomposition occurs at the orthographic-visual analysis stage and is not affected by lexical factors. We will now discuss the location and nature of the morphological decomposition at the early stages of visual-orthographic analysis and the nature of the effect morphology has on reading in neglexia in light of these findings.

### 4.1. The stage at which early morphological decomposition takes place

The results indicate that morphological decomposition occurs prelexically. The first clue for the pre-lexical application of the preliminary morphological decomposition comes from the main finding of this study: that the morphological structure of the target word had a clear effect on reading in neglexia: affixes were neglected significantly more often than root letters in the neglected side. Given that neglexia is a deficit at the pre-lexical visual-orthographic analysis stage (Caramazza and Hillis, 1990; Riddoch, 1990; Ellis and Young, 1996; Haywood and Coltheart, 2001; Vallar et al., 2010), the effect of morphology on reading in neglexia indicates that initial morphological analysis takes place at the orthographic-visual analysis stage.

Another clue for the stage at which the initial morphological decomposition is performed comes from the differential effect that perceptual factors (length and letter form) have on the neglect of affixes and root letters. These perceptual factors affected words ending with an affix but not words ending with a root letter. This finding also supports the idea that morphological decomposition occurs early, at the orthographic-visual analysis stage, at which perceptual factors are relevant.

Our findings also provide evidence that this prelexical decomposition is not affected by lexical and semantic factors from later stages, and that the effect on attention shift to the neglected side is not lexical. Most importantly, no difference was found between real roots and structurally-possible roots, and no difference was found between affixes that served as real affixes in the target word and potential affixes (like –er in *corner*); words with defective 2-letter roots ending with an affix consonant letter did not differ from words with three letter roots. These findings indicate that the decomposition is not guided by the lexicon.

In addition, there was no effect of the semantics of the target word on the erroneous response produced and no preference for errors that are semantically related to the target word. No difference was found between neglect errors that involved a derivational change and neglect errors that involved an inflectional change. Furthermore, neglect errors also did not preserve the morpho-lexical features of the words, such as lexical category, gender, and tense inflection. These findings indicate that lexical and semantic information and information on morpho-lexical features of the word are not yet accessible during this early stage of morphological analysis, and thus, that this decomposition occurs at a pre-lexical stage, without lexical feedback. This early morphological decomposition may take place in the orthographic-visual analyzer itself or in an orthographic input buffer that is holding all the information coming from the orthographic-visual analyzer until it is transferred to the lexical and sublexical routes.

These findings join studies from Hebrew concerning the active role of morphology in the lexical access of written words and the organization of the mental lexicon in this language, in the reading of skilled readers without dyslexia, and the centrality of the root in these processes and representations (Frost and Bentin, 1992; Katz and Frost, 1992; Frost et al., 1997, 2005; Deutsch et al., 1998, 2000).

Is early morphological decomposition part of visual analysis only in languages like Hebrew, where morphology plays a dominant role? Findings from normal reading in other languages also indicate that a preliminary morphological decomposition occurs before lexical access (Rastle et al., 2000, 2004; Longtin et al., 2003; Longtin and Meunier, 2005; Meunier and Longtin, 2007; Rastle and Davis, 2008; Beyersmann et al., 2011, 2013; Crepaldi et al., 2014) and that morphological structure even affects the reading of pseudowords, which are clearly not stored in the lexicon (Burani et al., 2006; Traficante et al., 2011).

Work on English by Rastle and Coltheart (2000); Rastle and Davis (2008); and McCormick et al. (2008) emphasize that morphological decomposition is a pre-lexical phenomenon that already operates at a very early stage of processing of complex words, and is based on orthographic analysis alone, regardless of lexical, semantic, or syntactic characteristics of the target word and its constituents. According to Meunier and Longtin (2007), the analysis that occurs at a preliminary stage of the processing of morphologically complex words is morpho-orthographic, and at the next stages of the reading process, information from higher processing stages is taken into consideration.

The fact that we studied the effect of morphology on reading using morphologically complex words that are constructed from a root, a derivational template, and an inflection allowed us to examine the effect of morphological decomposition that is independent of lexical contributions. To decompose a morphologically complex word in Hebrew, no access to a list of existing roots is needed. Decomposition can rely exclusively on the known structure of derivational templates, inflections, and the placeholders of the roots. This is probably what enabled us to see the very early effect of morphology on neglexia. In contrast, the decomposition of compounds, for example, is crucially dependent upon access to the words composing the compound, because structural knowledge cannot suffice, for example, to know where to segment a *cowboy* in English (or *Bauerngartenmischung, Wiesenblumensamen*, or *Sauerkirschsaft* in German). This explains the different findings of studies such as Mozer and Behrmann (1990), Behrmann et al. (1990), and Marelli et al. (2013), who studied compounds. Marelli et al., for example, found that the compound reading of their participants with neglexia was affected by two lexical variables: the type of compound (existing/non-existing) and the location of the head of the compound (right/left). They explained their findings in terms of the effect of the lexical information on the visual processing. Thus, whereas morphological decomposition occurs pre-lexically and is guided by the orthographic structure of the word (also according to Marelli, see for example Amenta et al., 2015), the analysis of compounds requires later stages and access to the lexicon, as compounds cannot be segmented solely based on a structural-orthographic analysis of the target word.

Furthermore, Arduino et al. (2002, 2003) found that the lexicality of the target affects the reading of some of their patients with neglect dyslexia. Such lexical contribution seems to be in effect after the stage into which we tapped in the current study: namely, when the information about the letters on the left side of the word is degraded, and this information is transmitted to the orthographic input lexicon, the lexicon can retrieve words that fit the partial information^8^. This happens, we believe, later than the effect that we described in the current study, where the attention shift has been affected by the morphological structure of the word, even before the lexicon was accessed.

### 4.2. The nature of the early morphological decomposition

What is the nature and mechanism of this prelexical morphological decomposition? One can consider two options: one is that the preliminary structural decomposition is based on identification of derivational templates and inflectional morphemes that are stored in the prelexical morphological analyzer; the other option is that the prelexical morphological analyzer holds a list of existing roots and the decomposition is based on the identification of an existing root in the target word.

Our findings indicate that the morphological analysis is based on a structural analysis of the morphological structure of the word, and does not rely on a list of existing roots (in line with Rastle and Coltheart, 2000; Rastle and Davis, 2008 and many others). Rather, the results suggest that it relies on information about morphological templates and affixes and their positions within the word. This conclusion is based on the finding that the early morphological decomposition is not sensitive to whether or not the root that can be structurally extracted from the target word is an existing root or not.

Further support for the structural nature of root extraction is that in words with defective roots composed of only two consonant root letters that end with an affix letter, the early analyzer mistook the final affix letter to be a third root letter. These findings indicate that lexical considerations and the existence of the root are not the basis for the early morphological decomposition. Theoretical considerations also disfavor an analysis at the orthographic-visual analyzer stage that is based on a list of existing roots, because such an assumption is not parsimonious and actually turns the visual analyzer into a lexicon.

The results indicate that the decomposition is guided by structural principles involving a search for three letters that can function as root letters structurally and not necessarily for an existing root. This finding corresponds with previous evidence concerning the structural quality of the process (Bentin and Feldman, 1990; Frost et al., 2000a,b; see also Rastle et al., 2004, for English, and Davis and Rastle, 2010 for a discussion).

Another indication for the way the structural decomposition is done comes from our finding that the presence of a prefix in words ending with a root letter did not raise the rate of neglect errors in left-sided neglexia in comparison with words without a prefix (e.g., mŠKL vs. ŠKL). Namely, the prefix letter is identified as an affix and is not counted as a root letter, and the search for three root letters continues. This mechanism led to similar neglect error rates in words of different lengths (3, 4, and 5 letters) ending with a root letter. As long as the first letters are identified as possible affixes, the morphological analyzer keeps shifting attention to the left until it identifies a three-letter root. For this procedure to occur, the morphological analyzer should have information about the possible affixes, and where in the word they can appear in their affix role.

Theoretically, this identification of “affix letters” can act in two different ways: letters that sometimes function as part of an affix may be identified as “morphological letters” and be neglected regardless of their role in the target word. Namely, the orthographic-visual analyzer may hold a list of letters that can be part of affixes, and these letters would be neglected even if they are part of the root in the target word. Alternatively, morphological decomposition may take place, according to some structural guidelines (looking for three letters of the root, taking into consideration which letters can play a morphological role of affixes), and then the letter would be judged according to its structural role in the target word and neglected only when it may structurally belong to an affix in the target word. Hebrew provides an excellent opportunity to determine between these two possibilities, as each letter that can be part of an affix can also be part of the root. For example, the final letter *m* can function as an affix in certain words (as part of the plural affix, or as the 3rd person plural possessive), and can thus be defined as a “morphological letter.” But this letter can also serve as part of the 3-consonant root. The findings of this study showed unequivocally that neglect errors took into account the morphological role of the letter in the target word. Namely, the letter was omitted only when it was part of an affix in the target word (structurally, though not necessarily lexically), whereas when it was (structurally, though not necessarily lexically) part of the root, it was not omitted. These findings indicate that the effect of morphology is not due to the orthographic-visual analyzer keeping a list of possibly-morphological letters, which are treated differently than root letters. Rather, these results indicate that the effect of morphology on neglexia is based on a morphological decomposition of the entire word, according to knowledge of inflectional and derivational templates and affixes and of the structure of Hebrew morphologically complex word. This analysis takes into account all the letters in the word and the complete morphological structure, and the structural role of each letter in the target word. Thus, an early, structural, morphological analysis already occurs before the neglect errors are made, leading to the neglect of letters in the neglected side only when they are analyzed structurally as an affix in the target word.

The picture that emerges from these findings and considerations is that during visual-orthographic analysis, the analyzer searches for three consonant letters that can function as the root letters. This search algorithm is based on the recognition of letters that have the potential to function as affixes, and where in the word they function as affixes (see also Crepaldi et al., 2010 for evidence from normal reading that the position of the affix in the word is taken into account, and discussion of this issue in Amenta and Crepaldi, 2012). If the affix letter appears in the relevant position within the target word, the morphological analyzer assumes it is part of an affix, and continues the search for three root letters. This is also the mechanism that protects root letters on the neglected side from omissions in neglexia.

### 4.3. Neglexia and the root

Reading errors in neglexia result from a deficit in attention allocation to one of the sides of the word. It is known that the spatial and visual framework can affect reading in neglexia. The current study showed that the morphological structure of the target word also affects reading in neglexia, as it modulates the allocation of attention to letters on the neglected side of the target word.

The morphological structure of the Hebrew language and orthography dictates the structure of the orthographic input lexicon, which is organized according to roots (Frost et al., 1997, 2005; Deutsch et al., 1998; Frost, 2012). This lexical organization, in turn, dictates the role of the orthographic visual analyzer—to extract the root that will enable access to the entry in the orthographic lexicon. Because of the important role of the root in lexical access, Hebrew readers, including Hebrew readers with neglexia, search for the letters of the potential root, and this search is a trigger for continued attention shift in neglexia.

The results suggest that morphological decomposition occurs pre-lexically, analyzing and identifying the template, affixes, and the possible root letters according to the structure of the target word. The analyzer identifies root letters and keeps them from omission. An attentional spotlight runs across the word, from right to left, in search for three root letters, and the attention shift in our neglexic participants was guided by this quest.

This quest for the three root letters also explains the finding that length affected words ending with an affix but not words ending with a root letter. When words ended with a consonant that was part of the root, the length of the word did not matter, and neglect errors did not occur more frequently in longer words. This is in contrast to words ending with an affix, for which a significant length effect was found. This indicates that as long as the quest for the three-letter root is not completed, attention shift to the left does not end, regardless of the word length. If the word includes an affix at the end of the word (i.e., on the left), after three root letters, the spotlight will stop after the three root letters have been identified, and the final affix letters will be neglected. By contrast, if an affix or even several affix letters appear in the word before all the root letters have been identified, and the word ends in a root letter, the spotlight will continue searching and reach the left end to recruit the 3 root letters, no matter how long the word is.

In this view, the effect of morphology on neglexia occurs very early, with the morphological structure directly affecting attention shift. The spotlight does not cease to shift attention to the left until the three root letters are identified. Once three root letters have been found, the spotlight is not “motivated” to search any further, and, given the attentional limitations affecting the left side, it stops, with a result of a neglect error.

This is in line with findings from the effect of the syntactic structure of sentences on reading in text-based neglect dyslexia. In a study of reading of sentences with different degrees of obligatoriness of the left component in the sentence, Friedmann et al. (2011) demonstrated that the syntactic structure of the sentence determined whether or not the readers keep shifting their attention toward the left side of the sentence, so that syntax served as a trigger for attention shift to the left of the sentence. A similar effect on neglect errors was also found in two-word compounds in Hebrew, where the right word included a morpho-phonological indication for the existence of another word on the left. This morpho-phonological indication increased the attention shift to the left word and reduced omissions of the left word (Friedmann and Gvion, 2014).

Quite similarly, at the word level, the current study shows that morphology serves as a trigger for attention shifting, and the visual analyzer continues to shift attention to the left side of the word until it identifies the three root letters.

### Conflict of interest statement

The authors declare that the research was conducted in the absence of any commercial or financial relationships that could be construed as a potential conflict of interest.

## Footnotes

^1^According to some approaches (cf., Sternberg and Friedmann, 2007, 2009) the output of the orthographic-visual analyzer is held in a short term graphemic memory component, the orthographic input buffer, until it is transferred to the orthographic input lexicon and the sublexical route.

^2^There are also seven bound morphemes in Hebrew, which are represented each by a single letter that is linearly affixed to the beginning of words, parallel to the, that, and, in, from, such as, and to in English. We do not test or discuss this type of morphology in the current paper (see Friedmann et al., 2015, in this research topic for findings regarding the morphological analysis of these prefixes in reading).

^3^In all the graphemic transcriptions throughout this article, root letters appear in capital letters and the rest of the letters are in lower case. The Hebrew words do not include this distinction in the orthography.

^4^In Hebrew, six letters protrude beyond the writing line—5 protrude downwards (, , , , ), and one upwards (). This visual salience did not seem to have an effect on neglect errors. Whereas all the participants made fewer neglect errors in words ending with a protruding letter, at the individual and group level, this was the result of most of the protruding letters being final-form letters, rather than their visual salience. When controlling for the final-form variable and the morphological letter variable, the visually-salient protruding letters are no longer more resilient to neglect errors that the other letters.

^5^In determining the sets of possible lexical neglect errors for each word for this analysis, we had to give homographs a special treatment. Homographic words can have different potentials for a neglect error that results in an existing word. For example, the word  (AHVh, ahava), means both the abstract noun *love*, and the verb *love-past-3rd-fem-sg*. Thus, a neglect error that changes  to the verb  (AHVo, ahavu, love-past-3rd-pl) can be analyzed in two different ways, depending on the meaning of the target homograph. If we consider ahava as a noun, the substitution is derivational, whereas if take it to be a verb, the substitution is inflectional. To determine which of the meanings to use in these cases, we collected the judgments of 50 native Hebrew speakers on the relative frequency of the meanings of each homograph. In cases in which there was an agreement of over 95% between judges on which meaning was more frequent, we used the meaning they agreed on. In cases the agreement rate was below 95%, we only used potential words that were common to all of the meanings. Homographic target words that were ambiguous between preserving and non-preserving feature were not included in the morpho-lexical feature preservation analysis.

^6^Among the target words there was only one word beginning with a potential affix, and thus we could not compare words beginning with a real affix and a potential affix. The word was removed from the calculations, thus, the category of words beginning with an affix only includes words beginning with a real affix.

^7^Recall that we created the stimuli so that a neglect error in each word can create another existing word. This applied both to words ending in affix letters and to words ending with root letters. Therefore, the larger rate of neglect errors in affixes cannot be ascribed to lexical completion or support from the lexical stage, as both kinds of words would receive equal support from the lexical level. As explained in Section 2.3.1, the analysis of the omission errors, for example, included only words in which the omission of the final letter, be it an affix or a root letter, creates another existing word.

^8^For this reason all the target words in the current study were selected so that a neglect error would create another existing word.

## References

[B1] Amenta S. Crepaldi D. (2012). Morphological processing as we know it: an analytical review of morphological effects in visual word identification. Front. Psychol. 3:232. 10.3389/fpsyg.2012.00232 22807919 PMC3395049

[B2] Amenta S. Marelli M. Crepaldi D. (2015). The fruitless effort of growing a fruitless tree: early morpho-orthographic and morpho-semantic effects in sentence reading. J. Exp. Psychol. Learn. Mem. Cogn. 41, 1587–1596. 10.1037/xlm0000104 25664370

[B3] Arad M. (2005). Roots and Patterns: Hebrew Morpho-Syntax. Dordrecht: Springer.

[B4] Arad M. Shlonsky U. (2008). Regularity and irregularity in the Hebrew verbal system, in Theoretical Hebrew Linguistics, ed Hatav G. (Jerusalem: Magnes, in Hebrew), 89–110.

[B5] Arduino L. S. Burani C. Vallar G. (2002). Lexical effects in left neglect dyslexia: a study in Italian patients. Cogn. Neuropsychol. 19, 421–444. 10.1080/02643290244000013 20957547

[B6] Arduino L. S. Burani C. Vallar G. (2003). Reading aloud and lexical decision in neglect dyslexia patients: a dissociation. Neuropsychologia 41, 877–885. 10.1016/S0028-3932(03)00015-0 12667524

[B7] Baayen R. H. Dijkstra T. Schreuder R. (1997). Singulars and plurals in dutch: evidence for a parallel dual-route model. J. Mem. Lang. 37, 94–117. 10.1006/jmla.1997.2509

[B8] Behrmann M. Moscovitch M. Black S. E. Mozer M. (1990). Perceptual and conceptual mechanisms in neglect dyslexia. Brain 113, 1163–1183. 10.1093/brain/113.4.1163 2397388

[B9] Bentin S. Feldman L. B. (1990). The contribution of morphological and semantic relatedness to the repetition effect at long and short lags: evidence from Hebrew. Q. J. Exp. Psychol. 42A, 693–711. 10.1080/14640749008401245 2287760

[B10] Beyersmann E. Castles A. Coltheart M. (2011). Decomposition during visual word recognition: evidence from masked transposed-letter priming. Psychon. Bull. Rev. 18, 937–942. 10.3758/s13423-011-0120-y 21713371

[B11] Beyersmann E. Dunabeitia J. A. Carreiras M. Coltheart M. Castles A. (2013). Early morphological decomposition of suffixed words: masked priming evidence with transposed-letter nonword primes. Appl. Psycholinguist. 34, 869–892. 10.1017/S0142716412000057

[B12] Biran M. Friedmann N. (2012). The representation of lexical-syntactic information: evidence from syntactic and lexical retrieval impairments in aphasia. Cortex 48, 1103–1127. 10.1016/j.cortex.2011.05.024 21798529

[B13] Burani C. Arduino L. Marcolini S. (2006). Naming morphologically complex pseudowords: a headstart for the root? Ment. Lex. 1, 299–327. 10.1075/ml.1.2.07bur

[B14] Burani C. Caramazza A. (1987). Representation and processing of derived words. Lang. Cogn. Process. 3, 217–227. 10.1080/01690968708406932

[B15] Butterworth B. (1983). Lexical representation, in Language Production: Development, Writing and Other Language Processes, Vol. 2, ed Butterworth B. (London: Academic Press), 257–294.

[B16] Caramazza A. Hillis A. E. (1990). Levels of representation, co-ordinate frames, and unilateral neglect. Cogn. Neuropsychol. 7, 391–445. 10.1080/02643299008253450

[B17] Caramazza A. Laudanna A. Romani C. (1988). Lexical access and inflectional morphology. Cognition 28, 297–332. 10.1016/0010-0277(88)90017-0 3359755

[B18] Chialant D. Caramazza A. (1995). Where is morphology and how is it processes? The case of written word recognition, in Morphological Aspects of Language Processing, ed Feldman L. B. (Hillsdale, NJ: Erlbaum), 55–76.

[B19] Coltheart M. (1984). Acquired dyslexias and normal reading, in Dyslexia: A global issue, eds Malatesha R. N. Whitaker H. A. (Lancaster: Martinus Nijhoff Publishers), 357–373.

[B20] Coltheart M. (1985). Cognitive neuropsychology and the study of reading, in Attention and Performance XI, eds Posner M. I. Marin O. S. M. (Hillsdale, NJ: Erlbaum), 3–37.

[B21] Coltheart M. Curtis B. Atkins P. Haller M. (1993). Models of reading aloud: dual-route and parallel-distributed-processing approaches. Psychol. Rev. 100, 589–608. 10.1037/0033-295X.100.4.589

[B22] Coltheart M. Rastle K. Perry C. Langdon R. Ziegler J. (2001). DRC: a dual route cascaded model of visual word recognition and reading aloud. Psychol. Rev. 108, 204–256. 10.1037/0033-295X.108.1.204 11212628

[B23] Crepaldi D. Morone E. A. Arduino L. S. Luzzatti C. (2014). Morphological processing of printed nouns and verbs: cross-class priming effects. J. Cogn. Psychol. 26, 433–460. 10.1080/20445911.2014.895007

[B24] Crepaldi D. Rastle K. Coltheart M. Nickels L. (2010). “Fell” primes “fall,” but does “bell” prime “ball?” Masked priming with irregularly-inflected primes. J. Mem. Lang. 63, 83–99. 10.1016/j.jml.2010.03.002

[B25] Davis M. H. Rastle K. (2010). Form and meaning in early morphological processing: comment on Feldman, O'Connor, and Moscoso del Prado Martín (2009). Psychon. Bull. Rev. 17, 749–755. 10.3758/PBR.17.5.749 21037177

[B26] de Lacy Costello A. Warrington E. K. (1987). The dissociation of visuospatial neglect and neglect dyslexia. J. Neurol. Neurosurg. Psychiatry 50, 1110–1116. 10.1136/jnnp.50.9.1110 3668560 PMC1032340

[B27] Deutsch A. Frost R. Forster K. I. (1998). Verbs and nouns are organized and accessed differently in the mental lexicon: evidence from Hebrew. J. Exp. Psychol. 24, 1238–1255. 10.1037/0278-7393.24.5.1238 9747532

[B28] Deutsch A. Frost R. Pollatsek A. Rayner K. (2000). Early morphological effects in word recognition in Hebrew: evidence from parafoveal preview benefit. Lang. Cogn. Process. 15, 487–506. 10.1080/01690960050119670

[B29] Diependaele K. Sandra D. Grainger J. (2009). Semantic transparency and masked morphological priming: the case of prefixed words. Mem. Cogn. 37, 895–908. 10.3758/MC.37.6.895 19679868

[B30] Ellis A. W. Flude B. M. Young A. W. (1987). “Neglect dyslexia” and the early visual processing of letters in words and nonwords. Cogn. Neuropsychol. 4, 439–464. 10.1080/02643298708252047

[B31] Ellis A. W. Young A. W. (1996). Human Cognitive Neuropsychology. Hillsdale, NJ: Erlbaum.

[B32] Ellis A. W. Young A. W. Flude B. M. (1993). Neglect and visual language, in Unilateral Neglect: Clinical and Experimental Studies, eds Robertson I. H. Marshall J. C. (Hillsdale, NJ: Erlbaum), 233–255.

[B33] Feldman L. B. Frost R. Pnini T. (1995). Decomposing words into their constituent morphemes: evidence from English and Hebrew. J. Exp. Psychol. 21, 947–960. 10.1037/0278-7393.21.4.947 7673870

[B34] Feldman L. B. Soltano E. G. (1999). Morphological priming: the role of prime duration, semantic transparency, and affix position. Brain Lang. 68, 33–39. 10.1006/brln.1999.2077 10433736

[B35] Friedmann N. Biran M. (2003). When is gender accessed? A study of paraphasias in Hebrew anomia. Cortex 39, 441–463. 10.1016/S0010-9452(08)70258-2 12870821

[B36] Friedmann N. Gvion A. (2003). Tiltan: Test Battery for Dyslexia. Tel Aviv: Tel-Aviv University [Hebrew].

[B37] Friedmann N. Gvion A. (2005). Letter form as a constraint for errors in neglect dyslexia and letter position dyslexia. Behav. Neurol. 16, 145–158. 10.1155/2005/635634 16410630 PMC5478848

[B38] Friedmann N. Gvion A. (2014). Compound reading in Hebrew text-based neglect dyslexia: the effects of the first word on the second word and of the second on the first. Cogn. Neuropsychol. 31, 106–122. 10.1080/02643294.2014.884059 24617530

[B39] Friedmann N. Gvion A. Nisim R. (2015). Insights from developmental and acquired letter position dyslexia on morphological decomposition in reading. Front. Human Neurosci. 9:143 10.3389/fnhum.2015.00143 PMC4490734 26190985

[B40] Friedmann N. Nachman-katz I. (2004). Neglect dyslexia in a Hebrew-reading child. Cortex 40, 301–313. 10.1016/S0010-9452(08)70125-4 15156788

[B41] Friedmann N. Tzailer-Gross L. Gvion A. (2011). The effect of syntax on reading in neglect dyslexia. Neuropsychologia 49, 2803–2816. 10.1016/j.neuropsychologia.2011.05.023 21679719

[B42] Frost R. (2012). Towards a universal model of reading. Behav. Brain Sci. 35, 263–279. 10.1017/S0140525X11001841 22929057 PMC3677812

[B43] Frost R. Bentin S. (1992). Reading consonants and guessing vowels: visual word recognition in Hebrew orthography, in Orthography, Phonology, Morphology, and Meaning, eds Frost R. Katz L. (Amsterdam: Elsevier), 27–44.

[B44] Frost R. Deutsch A. Forster K. I. (2000a). Decomposing morphologically complex words in a nonlinear morphology. J. Exp. Psychol. 26, 751–765. 10.1037/0278-7393.26.3.751 10855429

[B45] Frost R. Deutsch A. Gilboa O. Tannenbaum M. Marslen-Wilson W. (2000b). Morphological priming: dissociation of phonological, semantic, and morphological factors. Mem. Cogn. 28, 1277–1288. 10.3758/BF03211828 11219955

[B46] Frost R. Forster K. I. Deutsch A. (1997). What can we learn from the morphology of Hebrew? A masked-priming investigation of morphological representation. J. Exp. Psychol. 23, 829–856. 10.1037/0278-7393.23.4.829 9265076

[B47] Frost R. Kugler T. Deutsch A. Forster K. I. (2005). Orthographic structure versus morphological structure: principles of lexical organization in a given language. J. Exp. Psychol. 31, 1293–1326. 10.1037/0278-7393.31.6.1293 16393048

[B48] Giraudo H. Grainger J. (2000). Effects of prime word frequency and cumulative root frequency in masked morphological priming. Lang. Cogn. Process. 15, 421–444. 10.1080/01690960050119652

[B49] Giraudo H. Grainger J. (2001). Priming complex words: evidence for supralexical representation of morphology. Psychon. Bull. Rev. 8, 127–131. 10.3758/BF03196148 11340857

[B50] Haywood M. Coltheart M. (2001). Neglect dyslexia with a stimulus-centred deficit and without visuospatial neglect. Cogn. Neuropsychol. 18, 577–613. 10.1080/02643290042000251 20945229

[B51] Jackson N. Coltheart M. (2001). Routes to Reading Success and Failure. Hove: Psychology Press.

[B52] Katz L. Frost R. (1992). The reading process is different for different orthographies: the orthographic depth hypothesis, in Orthography, Phonology, Morphology and Meaning, eds Frost R. Katz L. (Amsterdam: Elsevier), 67–84.

[B53] Kempley S. T. Morton J. (1982). The effects of priming with regularly and irregularly related words in auditory word recognition. Br. J. Psychol. 73, 441–454. 10.1111/j.2044-8295.1982.tb01826.x 7171919

[B54] Kertesz A. (1982). Western Aphasia Battery. Orlando: Grune & Stratton.

[B55] Laudanna A. Burani C. (1985). Address mechanisms to decomposed lexical entries. Linguistics 23, 775–792. 10.1515/ling.1985.23.5.775

[B56] Longtin C.-M. Meunier F. (2005). Morphological decomposition in early visual word processing. J. Mem. Lang. 53, 26–41. 10.1016/j.jml.2005.02.008

[B57] Longtin C.-M. Segui J. Hallé P. A. (2003). Morphological priming without morphological relationship. Lang. Cogn. Process. 18, 313–334. 10.1080/01690960244000036

[B58] Lukatela G. Carello C. Turvey M. T. (1987). Lexical representation of regular and irregular inflected nouns. Lang. Cogn. Process. 2, 1–17. 10.1080/01690968708406349

[B59] Lukatela G. Gligorijeviæ B. Kostiæ A. Turvey M. T. (1980). Representation of inflected nouns in the internal lexicon. Mem. Cogn. 8, 415–423. 10.3758/BF03211138 7442544

[B60] Manelis L. Tharp D. A. (1977). The processing of affixed words. Mem. Cogn. 5, 690–695. 10.3758/BF03197417 24203287

[B61] Marelli M. Aggujaro S. Molteni F. Luzzatti C. (2013). Understanding the mental lexicon through neglect dyslexia: a study on compound noun reading. Neurocase 19, 128–144. 10.1080/13554794.2011.654222 22519604

[B62] Marshall J. C. (1984). Toward a rational taxonomy of the acquired dyslexias, in Dyslexia: A Global Issue, eds Malatesha R. N. Whitaker H. A. (The Hague: Martinus Nijhoff), 211–232.

[B63] Marslen-Wilson W. Tyler L. K. Waksler R. Older L. (1994). Morphology and meaning in the English lexicon. Psychol. Rev. 101, 3–33. 10.1037/0033-295X.101.1.3

[B64] McCormick S. F. Rastle K. Davis M. H. (2008). Is there ‘fete’ in ‘fetish’? Effects of orthographic opacity on morpho-orthographic segmentation in visual word recognition. J. Mem. Lang. 58, 307–326. 10.1016/j.jml.2007.05.006

[B65] Meunier F. Longtin C.-M. (2007). Morphological decomposition and semantic integration in word processing. J. Mem. Lang. 56, 457–471. 10.1016/j.jml.2006.11.005

[B66] Morton J. Patterson K. (1980). A new attempt at an interpretation, or, an attempt at a new interpretation, in Deep Dyslexia, eds Coltheart M. Patterson K. Marshall J. C. (London: Routledge and Kegan Paul), 91–118.

[B67] Mozer M. C. Behrmann M. (1990). On the interaction of selective attention and lexical knowledge: a connectionist account of neglect dyslexia. J. Cogn. Neurosci. 2, 96–123. 10.1162/jocn.1990.2.2.96 23972020

[B68] Nachman-katz I. Friedmann N. (2007). Developmental neglect dyslexia: Characteristics and direction for treatment. Lang. Brain 6, 75–90.

[B69] Newcombe F. Marshall J. C. (1981). On psycholinguistic classifications of the acquired dyslexias. Bull. Orton Soc. 31, 29–46. 10.1007/BF02658599

[B70] Patterson K. Wilson B. (1990). A ROSE is a ROSE or a NOSE: a deficit in initial letter identification. Cogn. Neuropsychol. 7, 447–477. 10.1080/0264329900825345125906058

[B71] Rastle K. Coltheart M. (2000). Lexical and nonlexical print-to-sound translation of disyllabic words and nonwords. J. Mem. Lang. 42, 342–364. 10.1006/jmla.1999.2687

[B72] Rastle K. Davis M. H. (2008). Morphological decomposition based on the analysis of orthography. Lang. Cogn. Process. 23, 942–971. 10.1080/01690960802069730

[B73] Rastle K. Davis M. H. Marslen-Wilson W. D. Tyler L. K. (2000). Morphological and semantic effects in visual word recognition: a time-course study. Lang. Cogn. Process. 15, 507–537. 10.1080/01690960050119689

[B74] Rastle K. Davis M. New B. (2004). The broth in my brother's brothel: morpho-orthographic segmentation in visual word recognition. Psychon. Bull. Rev. 11, 1090–1098. 10.3758/BF03196742 15875981

[B75] Riddoch J. (1990). Neglect and the peripheral dyslexias. Cogn. Neuropsychol. 7, 369–389. 10.1080/02643299008253449

[B76] Schreuder R. Baayen R. H. (1995). Modeling morphological processing, in Morphological Aspects of Language Processing, ed Feldman L. B. (Hillsdale, NJ: Erlbaum), 131–154.

[B77] Seidenberg M. S. McClelland J. L. (1989). A distributed, developmental model of word recognition and naming. Psychol. Rev. 96, 523–568. 10.1037/0033-295X.96.4.523 2798649

[B78] Shechther Y. (1965). ILAT: Israeli Loewenstein Aphasia Test. Ra'anana: Loewenstein Hospital Rehabilitation Center.

[B79] Soroker N. (1997). Hebrew Western Aphasia Battery. Ra'anana: Loewenstein Hospital Rehabilitation Center.

[B80] Sternberg T. Friedmann N. (2007). Developmental graphemic buffer dyslexia. Lang. Brain 6, 91–96.

[B81] Sternberg T. Friedmann N. (2009). Are there separate graphemic buffers for reading and writing? Lang. Brain 9, 105–117.

[B82] Taft M. (1979). Recognition of affixed words and the word frequency effect. Mem. Cogn. 7, 263–272. 10.3758/BF03197599 537502

[B83] Taft M. (1981). Prefix stripping revisited. J. Verbal Learning Verbal Behav. 20, 289–297. 10.1016/S0022-5371(81)90439-4

[B84] Taft M. (1994). Interactive-activation as a framework for understanding morphological processing. Lang. Cogn. Process. 9, 271–294. 10.1080/01690969408402120

[B85] Taft M. Forster K. I. (1975). Lexical storage and retrieval of prefixed words. J. Verbal Learning Verbal Behav. 14, 638–647. 10.1016/S0022-5371(75)80051-X

[B86] Taft M. Kougious P. (2004). The processing of morpheme-like units in monomorphemic words. Brain Lang. 90, 9–16. 10.1016/S0093-934X(03)00415-2 15172520

[B87] Traficante D. Burani C. (2003). Visual processing of Italian verbs and adjectives: the role of inflectional family size, in Morphological Structure in Language Processing, eds Baayen H. R. Schreuder R. (Berlin: Mouton de Gruyter), 45–64.

[B88] Traficante D. Marcolini S. Luci A. Zoccolotti P. Burani C. (2011). How do roots and suffixes influence reading of pseudowords: a study of Italian children with and without dyslexia. Lang. Cogn. Process. 26, 777–793. 10.1080/01690965.2010.496553

[B89] Vallar G. Burani C. Arduino L. S. (2010). Neglect dyslexia: a review of the neuropsychological literature. Exp. Brain Res. 206, 219–235. 10.1007/s00221-010-2386-0 20714712

[B90] Wilson B. Cockburn J. Halligan P. W. (1987). Behavioral Inattention Test. Suffolk: Thames Valley Test Company.

